# Effect of *Aedes aegypti* exposure to spatial repellent chemicals on BG-Sentinel™ trap catches

**DOI:** 10.1186/1756-3305-6-145

**Published:** 2013-05-20

**Authors:** Ferdinand V Salazar, Nicole L Achee, John P Grieco, Atchariya Prabaripai, Tolulope A Ojo, Lars Eisen, Christine Dureza, Suppaluck Polsomboon, Theeraphap Chareonviriyaphap

**Affiliations:** 1 Department of Entomology, Faculty of Agriculture, Kasetsart University, Bangkok, 10900, Thailand; 2 Department of Preventive Medicine and Biometrics, Uniformed Services University of Health Sciences, Bethesda, MD, 70814, USA; 3 Department of Mathematics, Statistics and Computer, Faculty of Liberal Arts and Science, Kasetsart University, Kamphaengsean, Nakhonpathom, 73140, Thailand; 4 Department of Microbiology, Immunology and Pathology, Colorado State University, Colorado, 80523, USA; 5 Research Institute for Tropical Medicine-Department of Health, FCC, 9:300 Research Drive, Alabang, Muntinlupa City, The Philippines

**Keywords:** *Aedes aegypti*, Spatial repellents, Screen house, Experimental huts, BG-Sentinel™ trap, Push-pull strategy, Thailand

## Abstract

**Background:**

An integrated approach to reduce densities of adult *Aedes aegypti* inside homes is currently being evaluated under experimentally controlled field conditions. The strategy combines a spatial repellent (SR) treatment (applied indoors) with the Biogents Sentinel^™^ (BGS) mosquito trap positioned in the outdoor environment. In essence, when combined, the goal is to create a push-pull mechanism that will reduce the probability of human-vector contact. The current study measured BGS recapture rates of *Ae. aegypti* test cohorts that were exposed to either SR or control (chemical-free) treatments within experimental huts. The objective was to define what, if any, negative impact SR may have on BGS trap efficacy (i.e., reduced BGS collection).

**Methods:**

*Aedes aegypti* females were exposed to SR compounds within experimental huts in the form of either treated fabric (DDT and transfluthrin) or mosquito coil (metofluthrin). Test cohorts were released within individual screen house cubicles, each containing 4 BGS traps, following SR exposure according to treatment. Two separate test cohorts were evaluated: (i) immediate release (IR) exposed from 06:00–12:00 hours and released at 12:00 hours and (ii) delayed release (DR) exposed from12:00–18:00 hours and released at 05:30 hours the following day. BGS recapture was monitored at 09:30, 13:30 and 15:30 hours and the cumulative recapture by time point quantified.

**Results:**

Exposure of *Ae. aegypti* females to either DDT or metofluthrin did not significantly impact BGS capture as compared to cohorts of non-exposed females. This was true for both IR and DR exposure populations. IR cohorts exposed to transfluthrin resulted in significantly lower BGS recapture compared to matched controls but this effect was primarily due to high mosquito mortality during transfluthrin trials.

**Conclusion:**

Our data indicate no more than minor and short-lived impacts (i.e., reduced attraction) on BGS trap catches following exposure to the pyrethroid compounds transfluthrin and metofluthrin and no change in recapture densities using DDT as compared to matched controls. These findings suggest a combined SR and BGS approach to vector control could function as a push-pull strategy to reduce *Ae. aegypti* adults in and around homes.

## Background

Dengue and dengue hemorrhagic fever occur in the tropics and subtropics with an estimated 2.5 billion people residing in areas where dengue is endemic [1]. Dengue viruses are transmitted primarily by *Aedes aegypti*, a day-biting mosquito that feeds and rests indoors and preferentially bites humans [2-5]. Despite years of public health efforts and research progress, an effective vaccine against dengue virus is not yet available. For this reason, disease prevention remains dependent on vector management and control strategies [1,4]. However, controlling *Ae. aegypti* has proven difficult due to its strong association with domestic and peridomestic human environments that harbor and sustain development sites (artificial containers) for the immatures. Furthermore, control of *Ae. aegypti* adults is commonly based on indoor and outdoor spraying of insecticides to reduce mosquito abundance and disrupt dengue virus transmission during outbreaks [6-8]. This is complicated by the worldwide rise and increasing impact of resistance of *Ae. aegypti* to commonly used insecticides, including in Thailand [9-12]. New approaches are urgently needed to improve our capacity to control this mosquito, especially targeting the adult stage in and around the home.

Push-pull strategies, combining a repellent with an attractant, have been effective in the control of some agricultural pests [13,14]. The mechanism underlying a push-pull system includes: (i) behavioral manipulation of the target species to repel or deter (push) them away from a resource (i.e., a crop) using stimuli that renders the resource unsuitable or unattractive and (ii) a device, for example a trap, through which the target species are removed from the environment (pull) [13-15]. Such an approach may also prove effective in the control of pathogen-transmitting mosquitoes, especially in and around the home where many vector-borne pathogens are predominantly transmitted in the developing world. One clear benefit of a push-pull system is that it can be effective in settings where insecticide resistance occurs. This is because the chemical doses that elicit sublethal behavioral responses, such as spatial repellency, are below that required for toxicity, thereby reducing insecticide resistance selection pressure while continuing to prevent human-vector contact [1,16-18].

Spatial repellents are defined as chemicals that function in the vapor phase to affect biting insects at a distance from the treatment source and can inhibit the ability of vectors to locate and track a host [19]. The vapor plume formed by the source of a spatial repellent creates a protective barrier extending to a certain radius from the source of the repellent chemical [20]. This has potential for protection of entire households. Moreover, continuous day and night protection can be provided through formulations that allow slow and continuous evaporation of the repellent substance. Continuous use of spatial repellents is expected to result in prevention of vectors from entering the treated space thereby prolonging times for the mosquito to locate hosts and/or resting places, thus increasing the likelihood of adverse environmental conditions, predation or other causes inducing mortality [21].

In the specific case of *Ae. aegypti* control, a trap or pull component may pose the greatest challenge within a push-pull system. Several trap designs have been commonly used for adult mosquito surveillance purposes [22,23]. However, many of these have not been satisfactory for *Ae. aegypti*[24-26]. The development of new, improved traps, such as the BG-Sentinel™ (BGS) and Zumba™ traps, provides an opportunity for improved entomological surveillance and possibly also control of *Ae. aegypti*[27-31] and *Ae. albopictus*[32,33]. The BGS trap targets the most important elements of *Ae*. *aegypti* host-seeking behavior by combining an olfactory cue (BGS Lure) with a visual cue (black and white contrast) to attract the mosquito. This trap has proven to be an effective tool for surveillance of *Ae. aegypti* adults, out-performing other collection devices such as the CDC backpack aspirator, the Fay-Prince trap, the Encephalitis Virus Surveillance trap and the Mosquito Magnet Liberty™ trap [29,30].

Based on these findings, the BGS trap was selected for evaluation in a push-pull *Ae. aegypti* control strategy currently under experimental evaluation. The push component uses spatial repellent chemicals that have been shown to deter Thai *Ae. aegypti* from entering homes (Ojo *et al.*, unpublished data). The BGS trap provides the pull component to remove repelled *Ae. aegypti* from the peridomestic environment thereby further reducing human-vector contact. Previous studies have confirmed that the BGS trap effectively removes *Ae. aegypti* from a controlled environment [34]. However, the effects of exposure to repellents, or sublethal doses of insecticides, on BGS trap collections have not been previously evaluated. For the pull component to be most effective, previous exposure to the spatial repellent being used to push vectors from entering homes should not substantially decrease the likelihood of the mosquito being trapped outdoors.

The objective of the current study was to define the effect of previous exposure of *Ae. aegypti* to spatial repellents in experimental huts on BGS trap efficacy. This information is important to define potential limitations in strategy success when both tools are used in combination. In addition, as a critical debate in the use of spatial repellents for vector control includes potential diversion or movement of repelled vectors to unprotected human hosts, findings will also provide insight as to how SR exposure may interfere with the host-seeking (i.e. attraction) response.

## Methods

### Study area and experimental huts

Studies were conducted near Pu Teuy (14°17′N, 99° 11′E), which is a small agricultural village (<1,500 inhabitants) located 150 km northwest of Bangkok in Sai Yok District, Kanchanaburi Province, Thailand. The village is situated in a mountainous area (420 m above sea level) and completely surrounded by dense primary forest, orchards and vegetable plantations. *Aedes aegypti* is prevalent in Pu Teuy village. The abundance of immatures in artificial water-holding containers is surveyed weekly, by the Thongpaphum District Clinic, and mosquito control interventions include distribution of organophosphate larvicide (temephos). Our experimental site is located >800 m from the closest indigenous home, creating a distance buffer for mark-release-recapture mosquito behavioral studies which exceeds the normal flight range (< 100 m) of *Ae. aegypti*[5,35]*.* The experimental huts used in the study have been previously described [36]. The huts mimic indigenous Thai homes in materials and dimensions, and are also used within the larger research program to evaluate *Ae. aegypti* entering and exiting behaviors as part of the development of the push-pull strategy [37].

### Mosquitoes

Immatures of *Ae. aegypti* were collected weekly from Pu Teuy village and reared to adults at the on-site field insectary. Female, nulliparous, 3–5 d old sugar-starved (i.e. reflecting a host-seeking physiological status) were used for the experimental trials. This age range and starvation treatment increased the probability that mosquitoes would respond to human host and BGS trap cues during evaluations. Mosquito test cohorts (control/treatment) were marked with unique colored fluorescent powder following previous dusting protocols [38].

One day pre-trial, cohorts (n = 50) were placed into individual ‘exposure cages’, mesh screen cages (26 × 26 × 30 cm), to: 1) facilitate transfer between huts and the screen house and 2) to prevent contact with treated surfaces so that chemical exposure would be based entirely on vapor phase particles. For each repellent treatment, two separate exposure cohorts were used: (i) an Immediate Release (IR) cohort exposed during 06:00–12:00 hours and then released into the screen house containing BGS traps at 12:00 hours and (ii) a Delayed Release (DR) cohort exposed during 12:00–18:00 hours and then released at 05:30 hours the following day, thus having a recovery period of nearly 12 h (with access to water soaked cotton pads). Individual screened cages were placed in the center of experimental huts according to exposure time. A matched control (i.e., chemical-free hut) was used simultaneously for each exposure trial.

### BG-Sentinel™ (BGS) trap

All BGS traps were baited with the BG-Lure and operated according to the manufacturer’s instructions. The trap consists of a collapsible container made of white plastic sack material. The top of the container is covered with white gauze cloth surrounding a black plastic funnel. This funnel is connected to a mesh catch bag that collects trapped mosquitoes. A 12 volt suction fan below the base of the funnel creates downward suction after connection to an external power source. The air is then pushed upwards passing through the gauze cover creating convection currents [27]. The contrasting black and white colors of the trap provide visual attraction. The accompanying BG-Lure consists of lactic acid, ammonia and caproic acid, compounds that are found in human sweat [39-41]. When the trap fan is operating, the air current carries the lure volatiles out through the gauze cloth cover into the surrounding environment. The BG-Lures were used within 4 months after opening per manufacturer’s recommendation.

### Chemical exposure

Two persons were present inside each of the experimental huts during trials to monitor coil burning and conduct collections for push-pull evaluations (Ojo *et al*. unpublished data). All test chemicals are USEPA registered and as such, have passed mammalian toxicology thresholds for human safety. Informed consent was conducted according to corresponding Uniformed Services University of the Health Sciences and Kasetsart University scientific and ethical review committee approvals.

The following repellent chemicals were evaluated in separate trials: 1) the organochlorine, DDT −1,1 Bis(4-chlorophenyl)-2,2,2- trichloroethane, (CAS 50-29-3,Sigma-Aldrich), 2) the synthetic pyrethroid, transfluthrin-2,3,5,6-tetrafluorobenzy(1R,3S)-3-(2,2-dichlorovinyl) 2,2dimethylcyclopropanecarboxylate (CAS118712-89-3, Bayer, AG) and 3) another synthetic pyrethroid, metofluthrin- 2,3,5,6-Tetrafluoro-4-(methoxymethyl)benzyl2,2-dimethyl-3-(prop-1-en-1-yl) cyclopropanecarboxylate (S.C. Johnson & Son, Inc). These chemicals represent standards in household mosquito control products (i.e., mosquito coils) or use within organized vector control campaigns (i.e. indoor residual spraying) and have been reported to have spatial repellent characteristics [16-18,21,42-55]. Although DDT has been prohibited for use in mosquito control programs in some areas, it was used here based on evidence of effectively controlling pests and mosquitoes transmitting malaria parasites and dengue virus [42,43,55].

Repellent treatments consisted of either chemical-treated fabric panels (DDT and transfluthrin trials) or a standard mosquito coil (metofluthrin trials). Matched control huts contained either chemical-free fabric (solvent only) or blank coil (coil without active ingredient). DDT was applied to fabric to mimic previous experimental hut studies evaluating its spatial repellent characteristics [17] and because this chemical is typically applied as an indoor residual spray to interior walls of houses [44]. Transfluthrin was similarly applied to fabric to match conditions employed for push-pull trials within the larger research project. Metofluthrin exposure was evaluated using a coil as this is a typical delivery format for the compound and volatile insecticides in general [45].

The preparation, treatment and positioning of DDT or transfluthrin-treated fabric inside experimental huts followed procedures previously described [17]. DDT and transfluthrin were applied to fabric panels 48 h pre-testing using solvent solution. The panels were air-dried under a chemical fume hood prior to storage at 4°C until used. Control fabric panels were treated with solvent alone and processed following the same procedure. Fabric panels were fitted onto metal mesh frames positioned along the interior walls of each hut using magnets [17,36]. One set of treated fabric panels was used for each experimental trial. DDT exposure was conducted using field application rate (FAR; 2 g *ai*/m^2^) at 75%, 50% and 25% surface area coverage (SAC). Evaluations were also performed using 25% SAC against 0.5 FAR (1 g *ai*/m^2^). Transfluthrin evaluations included 1.0 (40 μg *ai*/cm^2^), 0.5 (20 μg *ai*/cm^2^), 0.125 (5 μg *ai*/cm^2^), and 0.062 FAR (2.5 μg *ai*/cm^2^) using 25% SAC. The selected coverage during transfluthrin trials was based on data from experiments evaluating the push-pull system that showed 25% coverage to be as effective as coverage at 50% and 100% (Achee *et al.* unpublished data).

Metofluthrin coils representing high (0.0065% *ai*) and low (0.003% *ai*) doses were burned according to manufacturer’s recommendation within a metal dish positioned in the center of the hut. Coils were lit at 05:30 h, or 30 min before exposure cages were introduced, and were replaced at 12:00 h to ensure burning continued until 18:00 h, which represents the typical time range of expected *Ae. aegypti* biting period. The lag time following initial lighting allowed vaporization of the chemical and thereby distribution within interior air space. Both negative (no coil) and positive (coil without active ingredient) controls were used simultaneously in metofluthrin trials.

**Figure 1:**
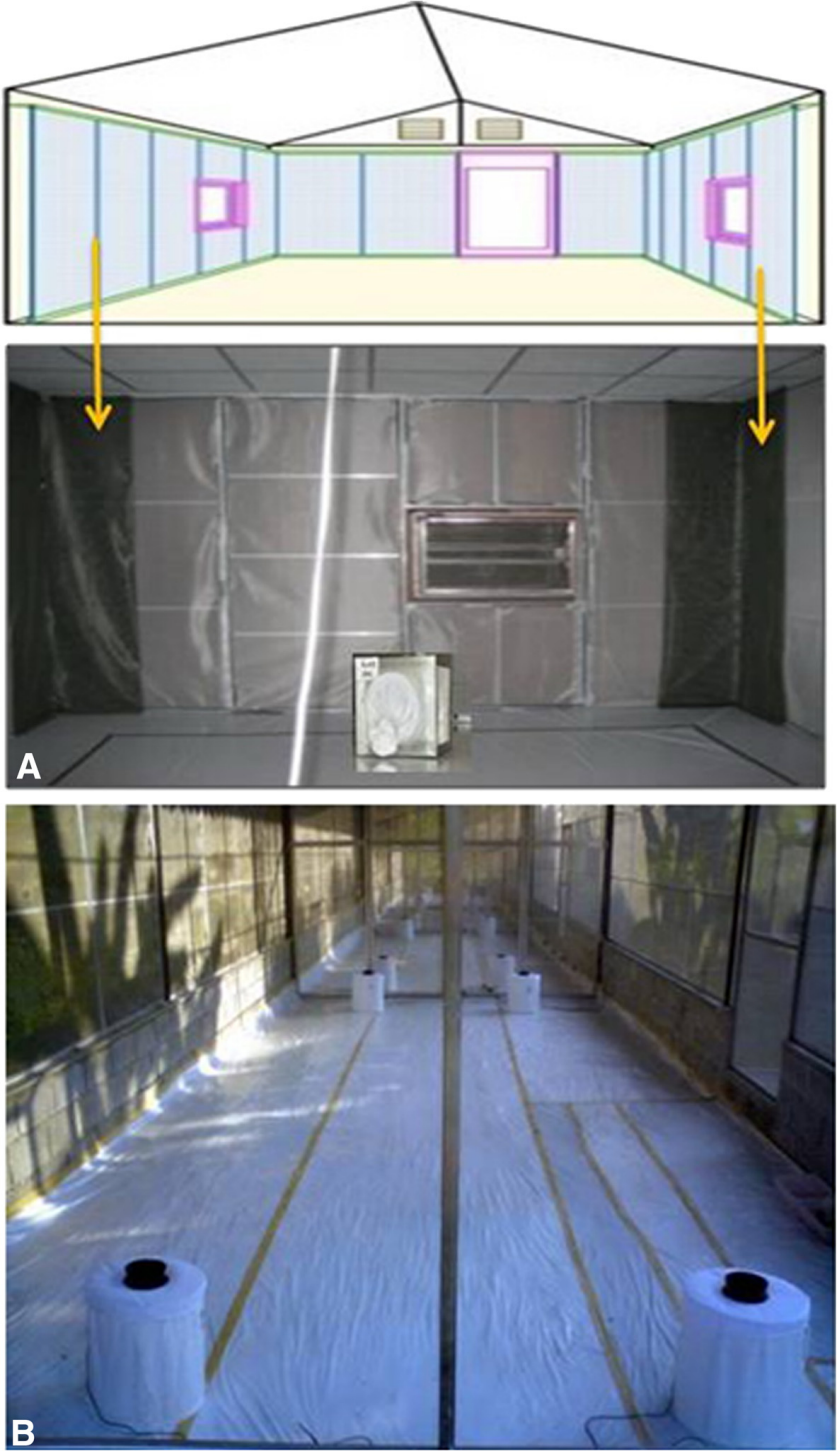
**(A) Positioning of treated material and exposure of *Aedes aegypti *in huts; (B) Mosquito trapping with BG-Sentinel™ traps in the screen house.**

On each trial day, one mosquito exposure cage (i.e. one test cohort) was placed in the center of each experimental hut (approximately 2 m from treated fabric panels and 1 m from burning coils) representing either control (chemical-free) or treatment (with spatial repellent) conditions (Figure 1). For trials with mosquito coils, an additional hut containing no coil was used as a negative control. A total of four replicates were performed for each chemical treatment.

### BGS capture evaluation

Post-exposure BGS captures were evaluated under semi-field screen house conditions. The screen house measures 4 m (width) × 3.5 m (height) × 40 m (length) and is located on-site with the experimental huts [34]. The screen house is subdivided into four 10 m long cubicles using metal partitions, each with a space volume of 140 m^3^ (Figure 1). This is similar to the volume inside and within 2 m outdoors of the experimental huts used for vector behavior studies at the field site [36] and is the expected approximate space volume within which *Ae. aegypti* primarily would make contact with an outdoor trap at a typical home in a dengue-endemic environment in Thailand [3,5].

Screen house cubicles were designated as control or treatment for evaluation of unexposed cohorts (chemical-free huts) and repellent-exposed cohorts, respectively. Within each cubicle, 4 BGS traps were operated simultaneously. The traps were monitored for mosquitoes based on sampling periods established from previous studies [35]. Those used for IR cohorts included: 13:30 and 17:30 hours Day of exposure (Day 1) and 05:30, 09:30, 13:30 and 17:30 hours Day following exposure (Day 2). Those used for DR cohorts included: 09:30, 13:30 and 17:30 hours Day 2 and 05:30, 09:30, 13:30 and 17:30 hours two days following exposure (Day 3).

Environmental parameters (temperature, relative humidity and light intensity) were recorded inside each cubicle using HOBO data loggers (HOBO U12-012 Model, Onset Computer Corporation, Bourne, MA). Baseline experiments were conducted to measure environmental variables among cubicles to ensure comparability before exposure studies were performed [34].

### Data analyses

Cumulative percentage BGS recapture of each test cohort were computed after correcting for the number of knocked down mosquitoes following exposure and prior to release. A Kruskal-Wallis statistical test was used to compare percentage recapture between treatment and control cohorts. The Mann–Whitney statistical test was used to compare cumulative BGS densities between IR and DR exposure cohorts. For all analyses, a p-value of 0.05 or less was considered statistically significant. Statistical analyses were performed in STATA 11.2 using the ranksum and kwallis syntax for Mann–Whitney test and Kruskal Wallis tests, respectively.

## Results

### DDT exposure

**Figure 2:**
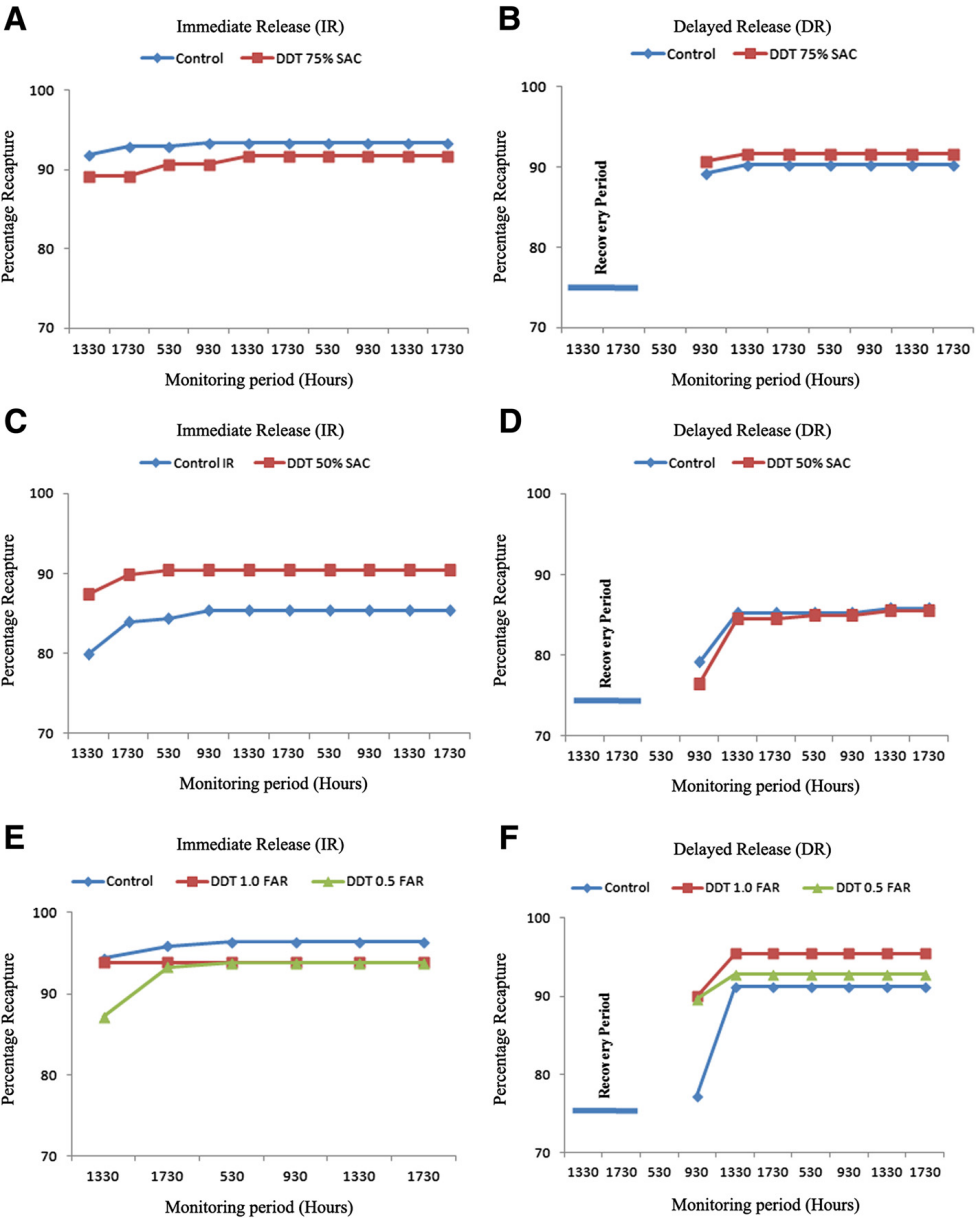
**Cumulative BG-Sentinel**^™^**trap recaptures for *Ae. aegypti *females in trials using immediate release or delayed release of mosquitoes previously exposed to DDT (2 g ai/m**^**2**^**) A and B - 75% SAC; C and D - 50% SAC) or DDT (2 g and 1 *ai*/m**^**2**^**) E and F - 25% SAC exposed populations.**

Cumulative BGS recapture, across application dose and SAC, ranged from 86-94% for DDT-exposed mosquitoes and 85-95% for control cohorts (Figure 2, Table 1). There was no significant difference in BGS recapture between DDT-exposed and control cohorts for either IR or DR populations (Figure 2, Table 1). This was true for evaluations using 75%, 50% and 25% SAC 1.0 FAR (2 g/m^2^) exposure conditions. Similar results were seen for trials using 25% SAC at 0.5 FAR (1 g/m^2^).

**Table 1:** **Cumulative BG-Sentinel™ trap catches for immediate release (IR)**^**1 **^**and delayed release (DR)**^**2 **^**trials with *Ae. aegypti***^**3 **^**exposed to DDT-treated fabrics**

		**Cumulative mean percentage (±SD) of released *Ae. aegypti *recaptured by time point**^**4**^													
**Surface area coverage**	**Release**^**5**^**/Treatments**	←‒‒‒**Day 1**‒‒‒‒‒→		←‒‒‒‒‒‒‒‒‒‒‒‒‒‒‒**Day 2**‒‒‒‒‒‒‒‒‒‒‒‒‒‒‒‒→				←‒‒‒‒‒‒‒‒‒‒‒‒‒‒‒‒‒**Day 3**‒‒‒‒‒‒‒‒‒‒‒‒‒‒‒‒‒‒‒→					**Mean day-time (12 hr) conditions**		
		**13:30 h**	**17:30 h**	**05:30 h**	**09:30 h**	**13:30 h**	**17:30 h**	**05:30 h**	**09:30 h**	**13:30 h**	**17:30 h**	N^6^	**Temp (°C)**	**RH (%)**	Light intensity (lx/ft^2^)
*75%*	**IRa**														
	Control	91.9a (±4.5)	92.9a (±3.5)	92.9a (±3.5)	93.4a (±3.5)	93.4a (±3.5)	93.4a (±3.5)	-	-	-	-	185/198	33.4	25.9	382.1
	DDT (2 g *ai*/m^2^)	89.2a (±5.3)	89.2a (±5.3)	90.7a (±4.1)	90.7a (±4.1)	91.8a (±3.8)	91.8a (±3.8)	-	-	-	-	178/194	31.9	28.6	331.6
	**DRa**														
	Control	-	-	-	89.3a (±4.8)	90.3a (±3.7)	90.3a (±3.7)	90.3a (±3.7)	90.3a (±3.7)	90.3a (±3.7)	90.3a (±3.7)	177/196	33.5	25.9	371.7
	DDT (2 g *ai*/m^2^)	-	-	-	90.7a (±5.2)	91.8a (±3.8)	91.8a (±3.8)	91.8a (±3.8)	91.8a (±3.8)	91.8a (±3.8)	91.8a (±3.8)	178/194	31.9	28.4	318.8
50%	**IRa**														
	Control	79.9a (±10.6)	83.9a (±4.9)	84.4a (±5.9)	85.4a (±5.5)	85.4a (±5.5)	85.4a (±5.5)					170/199	28.7	31.9	479.0
	DDT (2 g *ai*/m^2^)	87.4a (±9.6)	89.9a (±7.7)	90.4a (±7.7)	90.4a (±7.7)	90.4a (±7.7)	90.4a (±7.7)	-	-	-	-	178/198	29.7	69.1	454.5
	**DRa**														
	Control				79.2a (±10.8)	85.3a (±5.9)	85.3a (±5.9)	85.3a (±5.9)	85.3a (±5.9)	85.8a (±6.3)	85.8a (±6.3)	169/197	30.8	66.7	494.2
	DDT (2 g *ai*/m^2^)				76.5a (±11.1)	84.5a (±3.4)	84.5a (±3.4)	85.0a (±3.5)	85.0a (±3.5)	85.5a (±3.5)	85.5a (±3.5)	171/200	29.2	71.8	501.2
25%	**IRa**														
	Control	94.4a (±4.7)	95.9a (±4.5)	96.4a (±4.6)	96.4a (±4.6)	96.4a (±4.6)	96.4a (±4.6)	-	-	-	-	189/196	28.1	24.4	192.16
	DDT (2 g *ai*/m^2^)	93.9a (±3.0)	93.9a (±3.0)	93.9a (±3.0)	93.91a (±2.95)	93.9a (±3.0)	93.9a (±3.0)	-	-	-	-	185/197	27.4	26.0	126.2
	DDT (1 g *ai*/m^2^)	87.2a (±14.6)	93.3a (±3.9)	93.9a (±3.6)	93.9a (±3.6)	93.9a (±3.6)	93.9a (±3.6)					183/195	27.1	31.7	76.4
	**DRa**														
	Control	-	-	-	77.2a (±8.9)	91.2a (±3.7)	91.2a (±3.7)	91.2a (±3.7)	91.2a (±3.7)	91.2a (±3.7)	91.2a (±3.7)	176/193	28.4	25.3	205.5
	DDT (2 g *ai*/m^2^)	-	-	-	90.0a (±5.4)	95.5a (±2.6)	95.5a (±2.6)	95.5a (±2.6)	95.5a (±2.6)	95.5a (±2.6)	95.5a (±2.6)	190/199	27.8	26.6	135.2
	DDT (1 g *ai*/m^2^)	-	-	-	89.6a (±4.7)	92.8a (±4.11)	92.8a (±4.1)	92.8a (±4.1)	92.8a (±4.1)	92.8a (±4.1)	92.8a (±4.1)	181/195	27.5	32.8	85.6

^1^ Cohort exposed from 0600–1200 hours and released immediately afterwards.

^2^ Cohort exposed from 1200–1800 hours but released only after a holding period of 12 h.

^3^ 3–5 day old starved females.

^4^ Different lowercase letters in the same column indicate significant differences between mean recapture percentages for released females within release trial types (IR or DR; Kruskal-Wallis 95% confidence limit ) or between IR and DR trials (Mann-U Whitney Test, 95% confidence limit).

^5^ Different lowercase letters in the same column indicate significant differences between mean recapture percentages for released females between IR and DR trials (Mann-U Whitney Test, 95% confidence limit).

^6^Total recaptured /total released minus total knock down.

### Metofluthrin exposure

**Figure 3:**
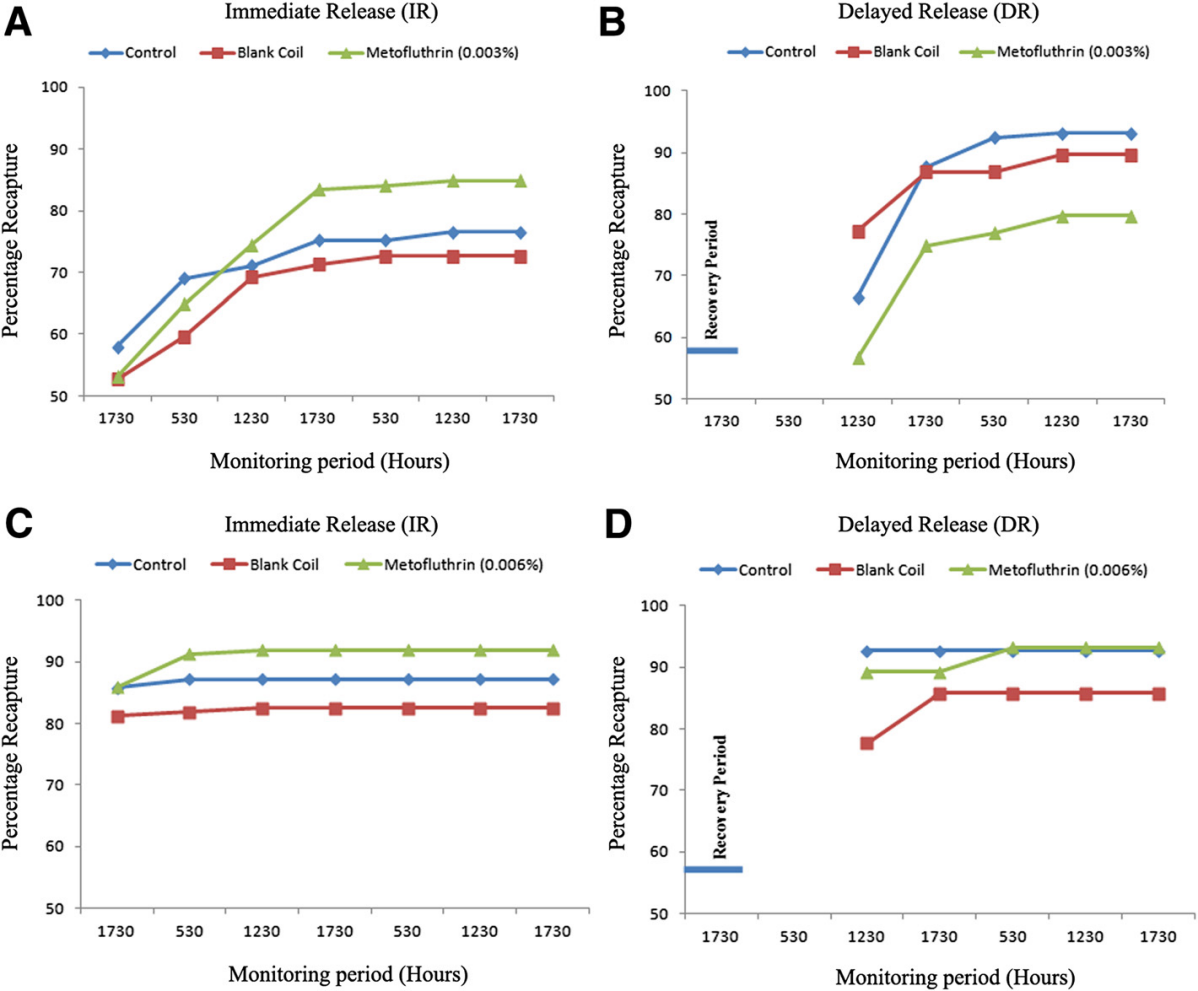
**Cumulative BG-Sentinel**^™^**trap recaptures for *Ae. aegypti *females in trials using immediate release or delayed release of mosquitoes previously exposed to metofluthrin: A and B - low dose (0.003%) and C and D - high dose (0.006%).**

There were no significant differences in BGS recapture rates among metofluthrin-exposed and both positive (blank coil) and negative control (no coil) cohorts for either IR or DR populations. This was true using both high (0.0065%) and low dose (0.003%) coils (Figure 3A-D, Tables 2). Cumulative recaptures ranged from 77-93% for negative controls, 73-90% for positive controls and a combined range of 80-93% for cohorts exposed to both low or high dose coils (Figure 3A-B, Table 2).

**Table 2:** **Cumulative BG-Sentinel™ trap catches for immediate release (IR)**^**1 **^**and delayed release (DR)**^**2 **^**trials with *Ae. aegypti***^**3 **^**exposed to metofluthrin coils**

		**Cumulative mean percentage (±SD) of released *Ae. aegypti *recaptured by time point**^**4**^										
	**Release**^**5**^**/Treatments**	**Day 1**	←‒‒‒‒‒‒‒‒‒‒‒‒‒‒‒**Day 2**‒‒‒‒‒‒‒‒‒‒‒‒‒‒‒‒‒→			←‒‒‒‒‒‒‒‒‒‒‒‒‒‒**Day 3**‒‒‒‒‒‒‒‒‒‒‒‒‒‒‒‒‒‒‒→				**Mean day-time (12 hr) conditions**		
**Dose**		**17:30 h**	**05:30 h**	**12:30 h**	**17:30 h**	**05:30 h**	**12:30 h**	**17:30 h**	N^6^	**Temp (°C)**	**RH (%)**	Light intensity (lx/ft^2^)
Low dose (0.00312%)	**IRa**											
	Control	57.9a (±5.6)	69.0a (±6.5)	71.0a (±6.3)	75.2a (±7.9)	75.2a (±7.9)	76.6a (±7.9)	76.6a (±7.9)	111/145	24.9	60.2	167.6
	Blank Coil	52.7a (±7.9)	59.6a (±13.6)	69.2a (±18.7)	71.2a (±6.3)	72.6a (±8.7)	72.6a (±8.7)	72.6a (±8.7)	106/146	25.2	59.9	177.9
	Metofluthrin	53.1a (±11.0)	64.8a (±8.2)	74.2a (±8.9)	83.5a (±1.3)	84.2a (±0.3)	84.8a (±1.1)	84.8a (±1.1)	123/145	24.1	61.8	66.6
	**DRa**											
	Control	-	-	66.4a (±18.8)	87.7a (±9.5)	92.5a (±2.6)	93.2a (±3.4)	93.2a (±3.4)	136/146	26.2	63.0	178.5
	Blank Coil	-	-	77.2a (±14.6)	86.9a (±3.7)	86.9a (±3.7)	89.9a (±1.7)	89.9a (±1.7)	130/145	26.3	62.7	173.9
	Metofluthrin	-	-	56.6a (±7.4)	74.8a (±10.3)	76.9a (±11.5)	79.7a (±13.8)	79.7a (±13.9)	113/143	25.2	65.7	61.5
High Dose (0.00625%)	**IRa**											
	Control	85.8a(±5.3)	87.2a (±5.8)	87.2a (±5.8)	87.2a (±5.8)	87.2a (±5.8)	87.2a (±5.8)	87.2a (±5.8)	123/148	28.1	65.6	167.6
	Blank Coil	81.2a (±8.9)	81.9a (±7.9)	82.6a (±6.9)	82.55a (±7.0)	82.6a (±6.9)	82.6a (±6.9)	82.6a (±6.9)	123/149	28.0	65.8	185.1
	Metofluthrin	85.9a (±6.0)	91.3a (±1.1)	92.0a (±1.9)	92.0a (±1.9)	92.0a (±1.9)	92.0a (±1.9)	92.0a (±1.9)	137/149	26.7	69.2	63.7
	**DRa**											
	Control	-	-	92.7a (±4.2)	92.7a (±4.2)	92.7a (±4.2)	92.7a (±4.2)	92.7a (±4.2)	144/150	28.0	65.8	194.8
	Blank Coil	-	-	77.7a (±1.6)	85.8a (±5.4)	85.8a (±5.4)	85.8a (±5.4)	85.8a (±5.4)	126/148	29.7	65.9	186.8
	Metofluthrin	-	-	89.2a (±5.4)	89.2a (±5.4)	93.2a (±3.0)	93.2a (±3.0)	93.2a (±3.0)	138/148	26.7	69.4	61.7

^1^ Cohort exposed from 0600–1200 hours and released immediately afterwards.

^2^ Cohort exposed from 1200–1800 hours but released only after a holding period of 12 h.

^3^ 3–5 day old starved females.

^4^ Different lowercase letters in the same column indicate significant differences between mean recapture percentages for released females within release trial types (IR or DR; Kruskal-Wallis 95% confidence limit ) or between IR and DR trials (Mann-U Whitney Test, 95% confidence limit).

^5^ Different lowercase letters in the same column indicate significant differences between mean recapture percentages for released females between IR and DR trials (Mann-U Whitney Test, 95% confidence limit).

^6^Total recaptured /total released minus total knock down.

### Transfluthrin exposure

**Figure 4:**
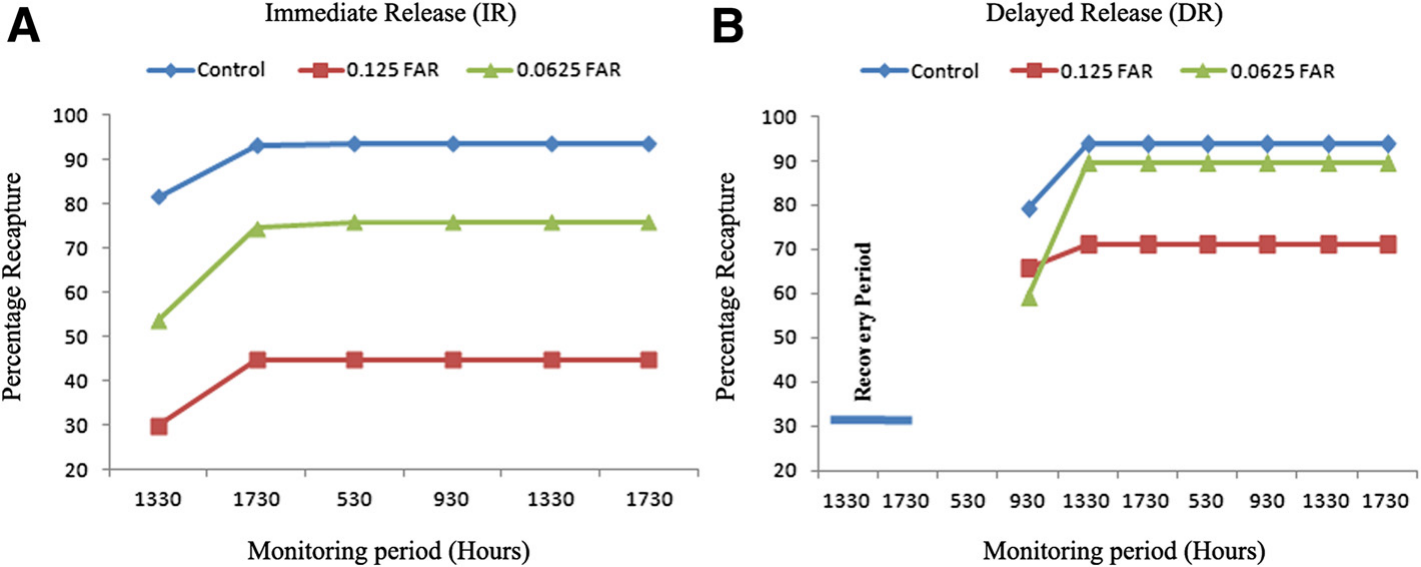
**Cumulative BG-Sentinel**^™^**trap recaptures for *Ae. aegypti *females in trials using immediate release (A) or delayed release (B) of mosquitoes previously exposed to transfluthrin.**

Exposure to transfluthrin at 1.0 FAR (40 μg *ai*/cm^2^) using 100, 50 or 25% SAC resulted in high mortality rates of *Ae. aegypti* cohorts. Mortality ranged from 95-100% and prevented BGS trap evaluations (Figure 4, Table 3). Trials using 0.125 FAR (5 μg *ai*/cm^2^) and 0.062 FAR (2.5 μg *ai*/cm^2^) at 25% SAC, however, did not have the same killing effect (23-69% mortality). Exposure to these treatment conditions resulted in significantly reduced BGS recapture for IR cohorts as compared to control; in contrast, there was no significant difference between BGS recapture of DR and control cohorts (Figure 4A-B, Table 3). Overall, BGS recapture was higher for DR as compared to IR cohorts (Figure 4A-B, Table 3). BGS trap catches for IR were reduced following exposure to the higher transfluthrin concentration (0.125 FAR), where only 45% of released females were recaptured as compared to 76% using 0.0625 FAR (*p = 0.01*).

**Table 3:** **Cumulative BG-Sentinel™ trap catches for immediate release (IR)**^**1 **^**and delayed release (DR)**^**2 **^**trials with *Ae. aegypti***^**3 **^**exposed to transfluthrin-treated fabrics**

	**Cumulative mean percentage (±SD) of released *Ae. aegypti *recaptured by time point**^**4**^													
**Release**^**5**^**/Treatments**	←‒‒‒‒‒**Day 1**‒‒‒‒‒→		←‒‒‒‒‒‒‒‒‒‒‒‒‒‒‒**Day 2**‒‒‒‒‒‒‒‒‒‒‒‒‒‒‒‒‒→				←‒‒‒‒‒‒‒‒‒‒‒‒‒‒‒‒‒‒‒**Day 3**‒‒‒‒‒‒‒‒‒‒‒‒‒‒‒‒‒‒→					**Mean day-time (12 hr) conditions**		
	**13:30 h**	**17:30 h**	**05:30 h**	**09:30 h**	**13:30 h**	**17:30 h**	**05:30 h**	**09:30 h**	**13:30 h**	**17:30 h**	N^6^	**Temp (°C)**	**RH (%)**	Light intensity (lx/ft^2^)
**IR**_**a**_ Control	81.8a (±11.1)	93.3a (±8.6)	93.8a (±7.6)	93.8a (±7.6)	93.8a (±7.6)	93.8a (±7.6)	-	-	-	-	180 /192	28.7	77.1	314.7
Transfluthrin (5 μg *ai*/cm^2^)	29.9c (±11.8)	44.8c (±18.7)	44.8c (±18.7)	44.8c (±18.7)	44.8c (±18.7)	44.8c (±18.7)	-	-	-	-	39/87	27.2	83.4	97.1
Transfluthrin (2.5 μg *ai*/cm^2^)	53.9b (±16.0)	74.7b (±9.3)	76.0b (±9.3)	76.0b (±9.3)	76.0b (±9.3)	76.0b (±9.3)					117/154	26.1	37.1	120.3
**DR**_**b**_														
Control	-	-	-	79.2a (±9.4)	93.9a (±4.3)	93.9a (±4.3)	93.9a (±4.3)	93.9a (±4.3)	93.9a (±4.3)	93.9a (±4.3)	185/197	28.0	78.6	308.3
Transfluthrin (5 μg *ai*/cm^2^)	-	-	-	65.6a (±18.3)	70.9a (±16.4)	70.9a (±16.4)	70.9a (±16.4)	70.9a (±16.4)	70.9a (±16.4)	70.9a (±16.4)	43/61	26.8	85.8	98.0
Transfluthrin (2.5 μg *ai*/cm^2^)	-	-	-	59.2a (±28.1)	89.6a (±4.6)	89.6a (±4.6)	89.6a (±4.6)	89.6a (±4.6)	89.6a (±4.6)	89.6a (±4.6)	92/138	25.7	37.7	126.8

^1^ Cohort exposed from 0600–1200 hours and released immediately afterwards.

^2^ Cohort exposed from 1200–1800 hours but released only after a holding period of 12 h.

^3^ 3–5 day old starved females.

^4^ Different lowercase letters in the same column indicate significant differences between mean recapture percentages for released females within release trial types (IR or DR; Kruskal-Wallis 95% confidence limit ) or between IR and DR trials (Mann-U Whitney Test, 95% confidence limit).

^5^ Different lowercase letters in the same column indicate significant differences between mean recapture percentages for released females between IR and DR trials (Mann-U Whitney Test, 95% confidence limit).

^6^Total recaptured /total released minus total knock down.

### Environmental parameters

No significant differences (*p > 0.05*) were found among mean daily temperature, relative humidity or light intensity variables measured within screen house cubicles designated as either control (for release of unexposed cohorts) or treatment (for release of repellent-exposed cohorts) for all trials (Tables 1, 2, 3).

## Discussion

The primary objective of the current study was to quantify the effects of exposure of *Ae. aegypti* to spatial repellent compounds on catch rates from a validated adult mosquito trap – the Biogents Sentinel™ (BGS) . The purpose was to generate critical information regarding how a spatial repellent may interfere with the efficacy of the BGS when the two tools are used in combination as a push-pull strategy. We specifically sought to determine: (i) if *Ae. aegypti* females exposed to spatial repellent chemicals (DDT, metofluthrin and transfluthrin) have a reduced likelihood of being captured with BGS traps (i.e., effect host-attraction) and (ii) if such an effect, should it occur, is immediate but short-lived or potentially latent.

Metofluthrin has been previously evaluated for repellency against *Ae. aegypti*[46,47], *Culex quinquefasciatus*[48,49] and *An. balabacensis*[48,49] and metofluthrin coils have been reported to significantly reduce landing counts of *Ae. aegypti*[46]. Transfluthrin is a fast acting insecticide that exhibits high volatility and knock down activity at high concentrations [50]. It is used in household products against various pest insects, such as mosquitoes, flies and moths. Evaluations have been conducted using transfluthrin to repel *Cx. quinquefasciatus*[51,52], *An. arabiensis*[53] and *Ae. albopictus*[54,56]. DDT has spatial repellent qualities as indicated in previous experimental hut studies [17,42,43].

The BGS trap has previously been validated as an effective tool for the monitoring and surveillance of the dengue virus vector *Ae. aegypti*[26-31]. However, the efficacy of the BGS trap to attract and catch (or pull) chemically-repelled or insecticide-exposed mosquitoes is not known. Many chemicals can elicit repellent behavioral responses in *Ae. aegypti* at doses well below those required for toxic outcomes [1,16,17], but the effects of such exposures on the mosquito’s host-seeking behavior are poorly understood. For another important dengue virus vector, *Ae. albopictus*, changes in both host-seeking and blood-feeding behaviors upon exposure to plant volatiles under laboratory conditions have been described [57]*. Aedes albopictus* females surviving exposure to geraniol, citral, eugenol, or anisaldehyde for 24 and 48 h all showed different degrees of reduction in host-seeking ability (e.g., increased times to reach a target location and to search for a suitable feeding site and insert the stylet). After 48 h of exposure to 0.250 μg/cm^3^ of anisaldehyde, 100% of the mosquitoes showed loss of host-seeking ability, through impacts on the time to host-seeking activation, orientation, probing and engorgement compared to unexposed controls. In another study, *Ae. aegypti* females were exposed to sublethal levels (LD_25_) of pyrethroid insecticides to evaluate the effects of the neurotoxicants 24 h post-exposure. A significant reduction in time of activation to flight was observed in mosquitoes exposed to deltamethrin and permethrin [58]. Similarly, excito-repellency studies using lower concentrations of deltamethrin showed that mated *Ae. aegypti* exhibited significant differences in escape responses with and without hosts present [59]. This type of knowledge is critical to define the expected efficacy of a BGS trap in a repellent focused push-pull strategy.

Holding female *Ae. aegypti* in experimental huts with DDT-treated fabric did not significantly impact subsequent BGS capture compared to non-exposed females, regardless of whether the exposed females were evaluated immediately following DDT exposure or following a 12 h recovery period in a repellent–free setting. Thus, there was no immediate or latent negative impact on host-seeking ability, as estimated by BGS trap catches, using this DDT exposure route. Similarly, mosquitoes exposed to metofluthrin coils with high (0.0065%) or low (0.003%) concentrations were as likely to be captured with the BGS traps as non-exposed control mosquitoes. Previous exposure to transfluthrin at 0.125 and 0.062 FAR resulted in significantly lower trap catches, compared to control mosquitoes, for mosquitoes released immediately following exposure but not for those allowed to recover for 12 h before BGS trap evaluation.

The comparison of results for mosquitoes released immediately following exposure versus those allowed to recover for 12 h before being released into the environment with the BGS traps indicate that this is a temporary phenomenon. This is suggestive of effects on sensory pathways used to detect host cues that resolve following the 12 h holding period. Similarly, Hao *et al.*[57] noted that a reduction in host-seeking ability in *Ae. albopictus* in the laboratory was reversible following recovery times that were dependent on chemical and concentration specific exposure conditions. However, we cannot rule out the possibility that the observed increase in host-seeking activity/trap catch rates for the delayed release females resulted, in part, from that they were only supplied with water during the recovery phase and thus were more motivated to locate a food source at the end of the recovery period.

Repellents have been shown to induce changes in responses of olfactory receptor neurons of female mosquitoes [60], specifically involving the grooved peg sensilla and sensilla trichodea, which are located on the mosquito antennae [61-64]. This neuronal activation disrupts the mosquito’s ability to detect host-seeking kairomones, components of human sweat and presumably also the BGS trap lure (BG Lure). The decline in sensitivity of *Ae. aegypti* to human odor as a result of repellent exposure might be a mechanism for the temporary suppression of host-seeking behavior [65], as seen for the IR mosquitoes exposed to transfluthrin. Most evidence published to date on the basis of action of repellent compounds (e.g., DEET) are clearly conflicting and support either hypotheses indicating that repellents mask odors by blocking their receptors or act as true odorants that seem to be avoided by pests. However, none of these studies refer to insecticides similar to those tested in the present study. Several insecticides have been described that induce hyperactivity at sublethal doses and even promote the avoidance of impregnated areas. However, no clear evidence exists to date to link these effects to those of known repellents acting on insect chemoreceptors. The specific mechanism of action behind the observed change in BGS recapture rates over time following exposure to transfluthrin, could not be addressed in the current study, but highlights the need to integrate laboratory and field evaluations as a model for translational research.

There are several study design biases that could have influenced our results. This includes innate differences in spatial repellent actives, such as volatility, and the fact that trials were performed independently at various times of year under varying temperatures that could also affect chemical volatility. Although the two treatment formats varied (treated fabric vs. coils), each format represented the typical exposure method that target vector mosquitoes would experience under operational implementation for these interventions. This allowed for a more accurate assessment of exposure effects of the spatial repellents as would be expected under natural conditions. In addition, the exposure methods used – where mosquitoes were held in screened cages placed inside experimental huts containing spatial repellent treatments (treated fabrics or coils) mimicked expected exposure routes for airborne repellent molecules where direct contact with treated surfaces does not occur. Despite these potential study design biases, it is clear that mosquito behavior is an area of research that will continue to be of high importance, especially as development of novel vector control tools are necessary in order to combat diseases such as malaria and dengue [66].

## Conclusion

Overall, data indicate that exposure of *Ae. aegypti* to the test repellents had no more than minor and short-lived impacts on BGS capture rates. This finding suggests the use of these repellents may not negatively impact the ability of the BGS to remove deterred *Ae. aegypti* adults from outdoor areas around treated homes. Deriving maximum benefits from an outdoor trap (pull) component within a repellent-focused combination push-pull system requires that previous exposure to a repellent chemical, used as the push component, will not substantially reduce trap efficacy to capture vectors from the peridomestic environment. We show here that exposure to DDT, metofluthrin or transfluthrin results in no more than minor and short-lived reductions in the efficacy of the Biogents Sentinel™ trap to recapture *Ae. aeygpti* females. However, using BGS recapture rates as a proxy for host-seeking, exposure to the highly volatile pyrethroid compound, transfluthrin, appeared to have some impact on test populations’ attraction or movement towards the trap immediately following exposure, (i.e., IR populations) based on reduced recapture rates but this effect was absent following a 12 h recovery period (i.e., DR populations). This delay may have significant impact on disease reduction, as several models have shown that the time period required for the mosquito to regain its ability to seek a host after repellent exposure likely increases the probability of mortality due to adverse environmental factors or predation [55]. This study has begun to elucidate an understanding of: (i) the selection of spatial repellent chemicals best suited for inclusion in a combined intervention system in which traps are required to function in removing repelled vectors from the peridomestic environment and (ii) the estimation of recovery time a vector may require to respond to an attractant source following repellent exposure.

## Competing interests

The authors declare no competing interests.

## Authors’ contributions

All the authors have contributed significantly to this study. FVS, NLA, JPG and TC contributed to conceptualization the study and wrote the manuscript. AP performed analysis the data. TAO, LE, CD and SP participated in its study and performed the coordination and helped the draft of the manuscript. All authors read and approved the final manuscript. Some of the authors are U.S. government employees. The opinions contained herein are the private views of the authors and are not to be construed as official or reflecting the views of the Department of Defense or the Uniformed Services University of the Health Sciences. Mention of specific commercial products does not constitute an endorsement or recommendation.
